# Parallel Advantage: Further Evidence for Bottom-up Saliency Computation by Human Primary Visual Cortex

**DOI:** 10.1177/03010066211062583

**Published:** 2022-01-13

**Authors:** Li Zhaoping

**Affiliations:** University of Tübingen, 28328 Max Planck Institute for Biological Cybernetics, Tübingen, Germany

**Keywords:** Saliency, primary visual cortex, V1 saliency hypothesis, visual search

## Abstract

Finding a target among uniformly oriented non-targets is typically faster when this target is perpendicular, rather than parallel, to the non-targets. The V1 Saliency Hypothesis (V1SH), that neurons in the primary visual cortex (V1) signal saliency for exogenous attentional attraction, predicts exactly the opposite in a special case: each target or non-target comprises two equally sized disks displaced from each other by 1.2 disk diameters center-to-center along a line defining its orientation. A target has two white or two black disks. Each non-target has one white disk and one black disk, and thus, unlike the target, activates V1 neurons less when its orientation is parallel rather than perpendicular to the neurons’ preferred orientations. When the target is parallel, rather than perpendicular, to the uniformly oriented non-targets, the target’s evoked V1 response escapes V1’s iso-orientation surround suppression, making the target more salient. I present behavioral observations confirming this prediction.

## Introduction

Where we should direct our attention or gaze is determined by both top-down (also called goal-dependent and endogenous) and bottom-up (goal-independent, stimulus-driven, and exogenous) factors (Treisman & Gelade, 1980; Theeuwes, 2010; Wolfe, 2021). For example, top-down factors guide our gaze to a page of a book we are reading, while bottom-up factors distract our gaze to an unexpected insect that suddenly appears beyond the book pages. Brain regions such as the frontal eye field (FEF) and parietal cortical areas are believed to be the neural basis for top-down control. However, the neural basis for bottom-up control is still hotly debated.

To limit the debate, define a visual location’s saliency as its ability to attract attention exogenously. A location can be salient by, for example, having a unique vertical bar among horizontal bars, a unique red item among green items, or a uniquely left-moving object among right-moving ones. Hence, saliency is general across many visual features (e.g., vertical orientation or red) and feature dimensions (e.g., orientation, motion, and color). This has motivated the idea that a saliency map to guide attention exogenously should reside in brain areas such as the FEF and parietal cortex, which contain retinotopic maps of the visual field and whose neurons are untuned to any specific visual features or feature dimensions (Koch & Ullman, 1985; Itti & Koch, 2001).

The V1 saliency hypothesis (V1SH), however, posits that saliency of a visual location is signaled by the highest response it evokes from V1 neurons whose receptive fields (RFs) cover that location, relative to the highest V1 responses to other locations. This is without regard to the preferred features (such as color and orientation) of the neurons (Li, 1999, 2002). For example, an orientation singleton, such as a unique vertical bar among horizontal bars, is salient because its evoked V1 response is higher than responses evoked by the background bars. The underlying neural mechanism is iso-feature suppression, whereby a V1 neuron’s response to visual input is typically suppressed by active neighboring neurons preferring similar input features (Knierim & Van Essen, 1992; Zhaoping, 2014). Hence, a neuron preferring horizontal orientation and responding to a horizontal background bar is, by iso-orientation suppression, suppressed by other horizontal-preferring neurons responding to nearby horizontal bars. In contrast, a vertical-preferring neuron responding to the unique vertical bar escapes the iso-orientation suppression, thereby giving a higher response. These V1 saliency signals are monosynaptically sent to the superior colliculus (SC, a midbrain area heavily involved in eye movement (Schiller, 1984)), allowing execution of an attentional shift to the most salient location. Iso-color suppression and iso-motion-direction suppression analogously make color singletons and motion-direction-singletons salient, respectively.

Various predictions of V1SH have been experimentally confirmed, see a review in Zhaoping (2014). They include the surprising phenomenon that an eye-of-origin singleton, such as a horizontal bar presented to the left eye among other horizontal bars presented to the right eye, captures attention automatically even when the singleton is task-irrelevant and indistinguishable to the observers from the background bars (Zhaoping, 2008). This singleton, whose unique eye-of-origin is visible to V1 but not to higher visual areas, evokes a higher V1 response due to iso-eye-of-origin suppression. V1SH has also been tested electrophysiologically in monkeys: V1 neurons’ responses to an orientation singleton are inversely correlated with the behavioral latencies to saccade to the singleton (Yan et al., 2018).

**Figure 1.**
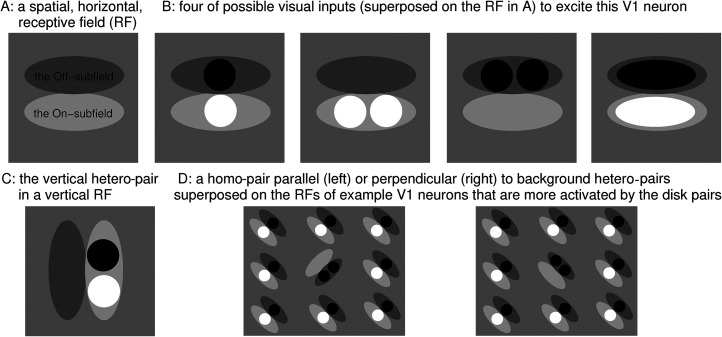
(A) Schematic of the Gabor-like, horizontally oriented, RF of a V1 neuron, with on- and off-subfields. (B) Activation of (A) neuron by a vertical hetero-pair, horizontal homo-pairs, and a horizontal Gabor. (C) Vertical hetero-pair in a vertical RF. The vertical hetero-pair activates a horizontal-tuned cell in (B) better than a vertical-tuned cell in (C). (D) Visual inputs resembling subparts of visual search stimuli in Figure 2 superposed on some example RFs of the V1 neurons that are more activated by the disk-pairs in these inputs. Panels (A)–(C) are adapted from Figure 2 of Zhaoping (2020).

**Figure 2.**
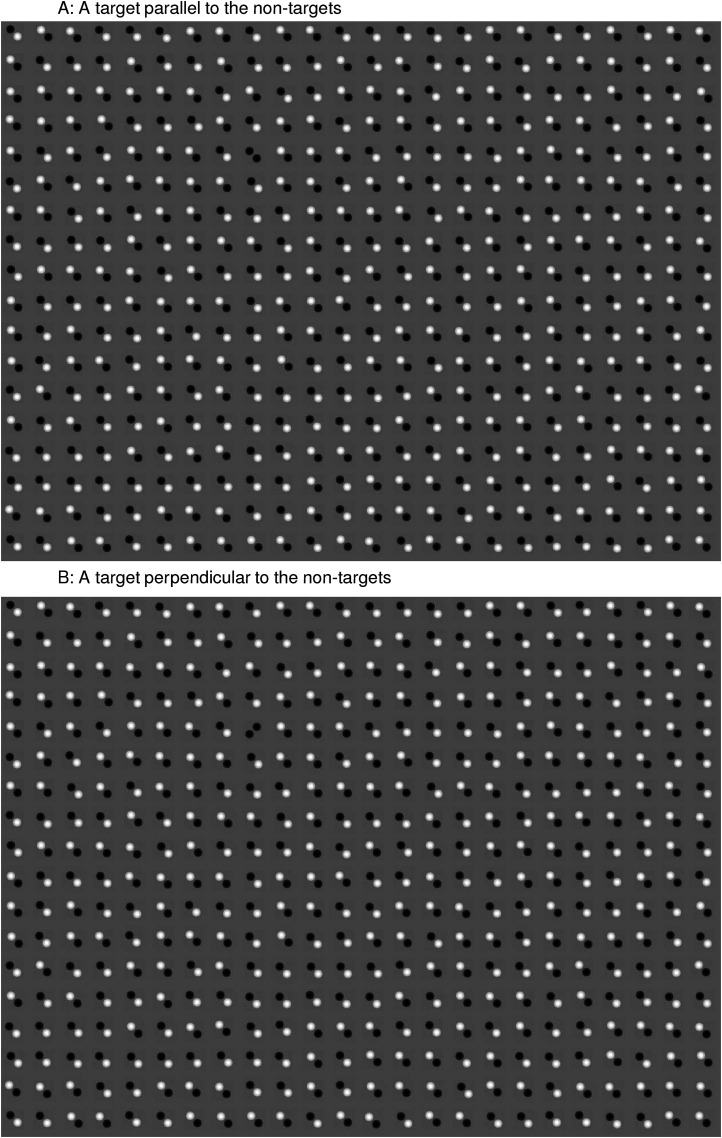
Two example search images. They differ only in the orientation of the target, a homo-pair of black disks, in the fifth row from the top and ninth column from the left. The non-targets are hetero-pairs of disks.

Here, we test a further surprising prediction of V1SH. We consider the search task of finding a target: a homo-pair of disks (two white disks or two black disks) in a background of hetero-pairs of disks (each made of one white disk and one black disk). The orientation of each disk-pair, target, or non-target, is that of the displacement between the two disks. Figure 1 shows that a hetero-pair better excites a V1 neuron preferring an orientation perpendicular rather than parallel to itself, since this is when the black and white disks, respectively, fall into the off- and on-subfields of the neuron’s RF. Smith et al. (2002) analyzed such a stimulus-response relationship in detail, showing that monkey’s V1 neurons indeed respond accordingly. Hence, when the background hetero-pairs are perpendicular to the target (homo-pair) (e.g., in Figure 2B), they mainly excite V1 neurons preferring the target’s orientation (see Figure 1D right), leading to iso-orientation suppression of the neuron responding to the target. In contrast, when the background hetero-pairs are parallel to the target (e.g., in Figure 2A), the V1 neuron responding to the target escapes this suppression (see Figure 1D left). Hence, V1SH predicts that this target is less salient when perpendicular rather than parallel to the non-targets. By contrast, higher brain areas are likely to define the orientation of the non-targets at the more symbolic level of the displacement between the two disks in each pair, and so predict that the target would be less salient when parallel rather than perpendicular to the non-targets as would be conventional in the visual search literature (Wolfe, 2021).

## Experimental Methods and Results

### Methods

The search images (as in Figure 2) contained 24 columns and 18 rows of disk pairs. They were displayed on a Sony cathode ray tube of resolution $1264 \times 948$ pixels, spanning $38.3\, {{\rm cm}} \times 29.1\, {{\rm cm}}$ or $48.0^\circ \times 37.4^\circ$ in visual angle at a 43 cm viewing distance in a dim room.

Each trial started with a central fixation cross on a gray background for 0.7 second, followed by 0.2 second without the fixation cross before a search image appeared waiting for an observer’s response. Observers had to fixate on the cross until the search image appeared, and then could freely move their gaze and press a left or right button as quickly as possible to report, respectively, a target in the left or right half of the display. They started the next trial with another button press. Right after several practice trials, each observer performed 400 testing trials and typically took one short break in the middle. The average durations ( $\pm$ standard deviations) to complete the first 200 testing trials, the second 200 testing trials, and all the 400 testing trials (including break times) were $18.8\pm 4.8$ , $13.9 \pm 1.9$ , and $36.2 \pm 8.0$ minutes, respectively.

Excluding the central four columns in the search array, the target was randomly and equally likely at any location within 9 columns and 7 rows from the display center, making it between about $5.4^\circ$ and $21.7^\circ$ from the display center. In each trial, the target was also randomly and equally likely to be a white or black homo-pair and oriented $45^\circ$ clockwise or counter-clockwise from vertical. Meanwhile, the uniformly oriented non-targets were randomly and equally likely to be parallel or perpendicular to the target. The black disk in each non-target was randomly and independently the upper or lower one with equal chance. The gray background had luminance $L_o = 44\, {{\rm cd}/m}^2$ . Each disk’s diameter was $D=15$ pixels, two disks in a pair were $R=18$ pixels apart center-to-center, a disk pair spanned $1.33^\circ \times 0.61^\circ$ at the display center. With $D\approx R$ , the most activated V1 neurons by each homo- or hetero-pair should prefer spatial frequency (SF) ${\sim \;}0.69$ cycles/degree. This SF is within the range of preferred SFs of monkey V1 neurons for each target eccentricity (Gattass et al., 1987) from the display center. Within a disk, each pixel at a distance $r$ from the disk's center had a luminance deviation from the gray background by about $L_o \cdot \exp \lpar -r^2/\lpar 2\sigma ^2\rpar \rpar $ with $\sigma = 5.4$ pixels. The location of each disk pair was randomly jittered, horizontally and vertically, from the regular $24\times 18$ grid such that two neighboring pairs were between 49 and 57 pixels (mean 52.6 pixels) apart center-to-center horizontally and vertically.

To test whether reaction times (RTs) for different target conditions differed significantly from each other, paired permutation tests (rather than $t$ -tests) across observers on their trial-averaged quantities are used, so that no assumptions need to be made regarding whether the RTs are normally distributed. Trials with RT $\lt 0.2\, {\rm second}$ or a wrong button press constituted no more than 2% of the trials for each observer, and are excluded from the data analysis. The qualitative results are insensitive to whether the RT outliers are removed, and are also insensitive to whether the trial RTs are transformed to their logarithms (to make the distribution of the data closer to a normal distribution) for the data analysis.

### Results

**Figure 3.**
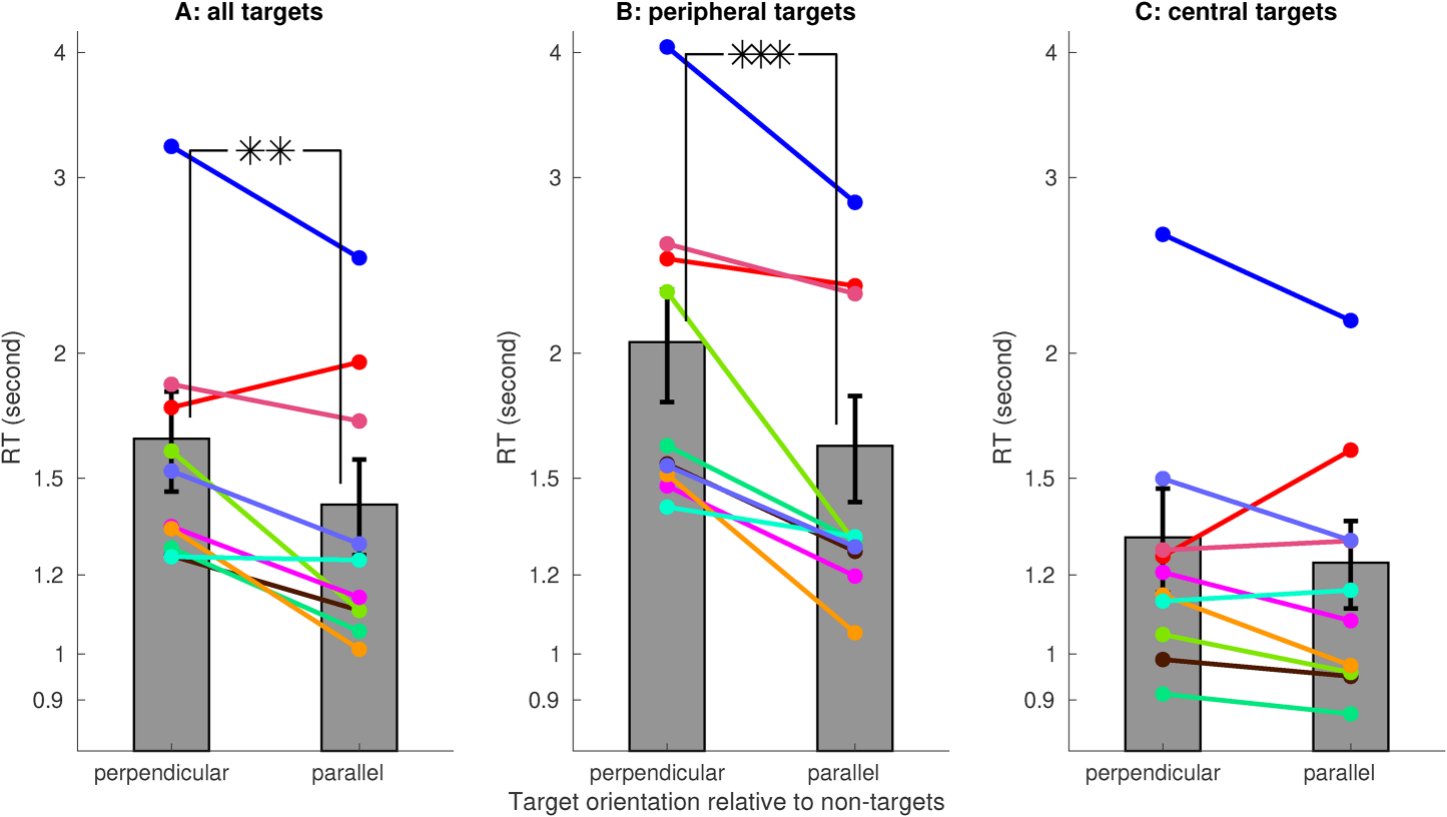
Reaction time (RT) to find a target was shorter for targets parallel rather than perpendicular to the non-targets. A target that is more or less than its average distance ( $14^\circ$ ) from the display center is termed “peripheral” or “central,” respectively. In the current and next figures, data bars mark the average RTs across $n=10$ observers (error bars are the standard errors); RTs of individual observers are visualized by individually colored dots linked by the correspondingly colored lines; two data bars linked by one, two, or three ’*’s indicate that their RT difference is significant with $p$ values $.05\lt p \le.01$ , $.001 \lt p\le.01$ , or $p\le.001$ , respectively.

**Figure 4.**
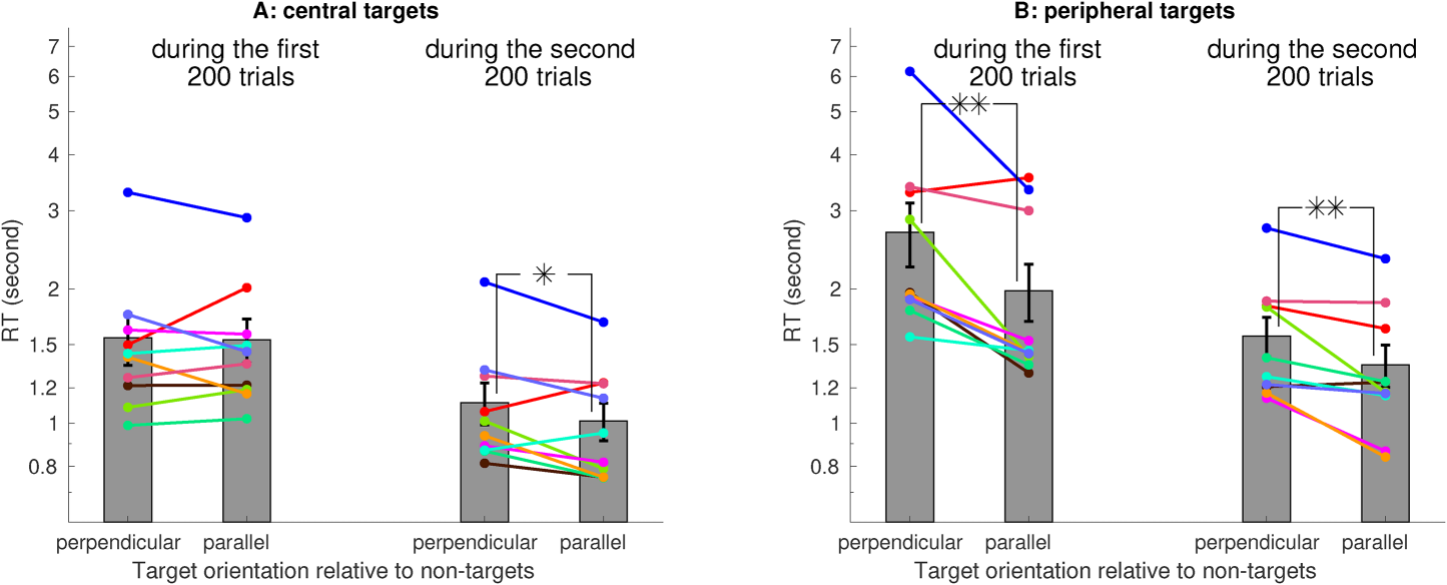
Learning effects. Each observer had several practice trials right before the 400 testing trials. The saliency effect, manifested as a shorter RT for the parallel than the perpendicular target, was significant for the peripheral but not the central targets in the first 200 testing trials, and became significant for the central targets in the second 200 testing trials.

Averaged across 10 observers (eight of them were naive and one of them was the author), the RT was significantly shorter when the target was parallel rather than perpendicular to the non-targets (Figure 3A), confirming the V1SH prediction. This RT difference was mainly due to the peripheral target trials, in which the target was further from the center of the visual display (Figure 3B). However, for the central target trials (i.e., nonperipheral target trials), this RT difference was insignificant (Figure 3C). As observers progressed toward the second 200 of their 400 trials, RTs decreased, and the RT difference became significant for the central target trials (Figure 4A). Meanwhile, for the peripheral target trials, this RT difference was significant already in the first 200 trials (Figure 4B).

## Summary and Discussion

Whether a target was parallel or perpendicular to the non-targets was unpredictable in each trial and irrelevant to the search task in the current study. Hence, the parallel target was found faster purely by bottom-up, or exogenous, factors. Indeed, we have defined saliency as the strength of attentional attraction by exogenous factors (Zhaoping, 2014). Since the target and non-targets, respectively, excite V1 neurons preferring orientations parallel and perpendicular to themselves (Smith et al., 2002), the preferred orientations of the activated V1 neurons, rather than the figure orientations (i.e., the main axis) of symbolic objects (the disk pairs), dictate saliency.

Among primate brain areas such as FEF , lateral intraparietal (LIP) area, superior colliculus (SC), and V1 that have been considered for saliency computation, V1 is most selective to the orientation feature, particularly for the sort of small disk pairs we used in this study. Neurons in FEF and LIP are typically untuned to visual features unless the features are relevant for a task on which the animal has been extensively trained (Bichot et al., 1996; Freedman & Assad, 2006). In monkey SC neurons, traditional studies suggest that tunings to visual features are absent or very weak except in extensively trained monkeys (Schiller, 1984; Horwitz & Newsome, 2001). More recent works (Chen & Hafed, 2018; Chen et al., 2018) reported modest feature tunings to orientation and spatial frequency in monkey SC neurons, although these forms of tuning are much weaker than those in V1 neurons.

To read out a V1 saliency map for an attentional shift to the most salient location by the winner-take-all computation, any feature signals (which are irrelevant for implementing a gaze or attentional shift) in V1 responses should be degraded and eliminated progressively along the read-out route (Zhaoping, 2014, 2016). The weaker feature selectivity in SC compared to V1 is consistent with the idea that SC, which has a longer response latency than V1 (White et al., 2017), inherits and degrades feature selectivity from V1 when reading out the saliency map. This selectivity degradation could even start in V1’s layer 5 neurons which project to SC (see Zhaoping, 2014 and 2016 for discussions). Indeed, in mice (in which neural types are more easily differentiated experimentally than in monkeys), such layer 5 neurons have weaker feature tunings than other layer 5 neurons (Lur et al., 2016). Parietal and frontal areas can also inherit saliency signals from V1 to combine them with top-down factors for controlling attention (Bisley & Goldberg, 2011; Shomstein, 2012).

This study adds to previous behavioral, electrophysiological, and neural imaging investigations that confirm V1SH predictions (Zhaoping, 2014; Yan et al., 2018). For our current stimuli, V1SH does not precisely predict whether the RTs (for a parallel or perpendicular target) should be insensitive to the number of non-targets, that is, the search set size. Hence, measuring the set size effect, interesting though it is for research in visual search, would neither support, nor falsify, V1SH. One may also ask, if the target is a hetero-pair while non-targets are uniformly oriented and randomly mixed black homo-pairs and white homo-pairs (analogous to our current design), whether a parallel target should yield a shorter RT than a perpendicular target by the same V1 mechanisms. The answer is unclear since, unlike a hetero-pair whose average luminance (locally) matches that of the gray background, a homo-pair is brighter or darker. Consequently, a random array of black and white homo-pairs will create a luminance interference that, analogous to the color interference in orientation target search (Snowden, 1998; Zhaoping & Snowden, 2006; Zhaoping & May, 2007), should overwhelm any saliency effects by the target’s orientation. Therefore, such a study would also be unable to support or falsify V1SH.

However, future studies could test other relevant predictions. For example, consider an orientation-tuned V1 neuron activated by an optimally oriented homo-pair in its RF. Surround suppression from uniformly oriented hetero-pairs outside the RF should be stronger when these hetero-pairs are perpendicular rather than parallel to the homo-pair. For another example, if the two disks in our hetero-pair are both sufficiently lighter or both sufficiently darker than the gray background, then the preferred orientation of the most activated V1 neurons should be parallel rather than perpendicular to the hetero-pair. Using such hetero-pairs for our search task, and making sure that all disks (from the target and non-targets) are all lighter or all darker than the gray background to prevent the kind of luminance interference mentioned above, V1SH predicts that a perpendicular (homo-pair) target should be found more quickly than a parallel target (Jeremy Wolfe, private communications).

Our observers reported that the search was effortful in the initial trials and that it became increasingly easier and reflexive in later trials. This learning may be related to similar learning effects in an orientation singleton search task in monkeys observed in Yan et al. (2018), although the number of trials for the monkeys was much larger. The monkeys’ learning was reflected in the correlation between shorter behavioral RTs (to saccade to the target) and higher V1 responses to the orientation contrast between the target and (uniformly oriented) non-targets (Yan et al., 2018). During the initial trials, this correlation was mainly with the V1 responses 100–200 ms after the search stimulus onset (and before the saccade); and the correlation with the initial V1 responses (40–60 ms after the stimulus onset) was insignificant except for the more salient targets. As monkeys’ RTs decreased through practice, the correlation with the later V1 responses decreased, whereas the correlation with the initial V1 responses increased and became significant even for the less salient targets. The substantial presence of the initial V1 responses (to the orientation contrast) was regardless of whether the monkey was doing the search task, whereas that of the later responses was only during the task (Yan et al., 2018), suggesting that the early and later V1 responses reflect bottom-up and top-down mechanisms, respectively. The learning, by the monkeys and by our observers, perhaps involves coming to rely more on the bottom-up saliency signals and less on the top-down control for the task. The 40 ms latency (of the initial V1 responses to the orientation contrast of the target) is too short for the V1 saliency signals to arise from feedback from downstream brain areas such as FEF, LIP, or SC.

Like previous studies (Zhaoping & Guyader, 2007; Nuthmann et al., 2021), this study also illustrates the central-peripheral dichotomy that peripheral and central vision are mainly for looking (to select where to shift attention to) and seeing (object recognition), respectively (Zhaoping, 2019). The contrast between the saliency effects (i.e., shorter RTs to find a parallel target) in our central versus peripheral targets is consistent with the idea that saliency processes are stronger for more peripheral visual locations (Zhaoping, 2014). A target closer to the fovea is more likely to lie within the attentional spotlight and so to be subject to top-down factors (Zhaoping, 2019) beyond bottom-up saliency. A target in the peripheral visual field is more vulnerable to visual crowding, so that it attracts gaze mainly by its saliency rather than by a recognition of its shape. Since saliency is the strength to attract attention exogenously, saliency-guided gaze shifts should in principle occur without recognizing the object at the saccade destination. In practice, this occurs at least in the case of gaze and attentional capture by the eye-of-origin singleton even though observers could not distinguish this singleton from other items in the visual field (Zhaoping, 2008, 2012). Our observed learning, which particularly increased the saliency effects in our central target trials, enabled our observers to employ more looking rather than seeing processes for this task.
